# A thyroid hormone network exists in synovial fibroblasts of rheumatoid arthritis and osteoarthritis patients

**DOI:** 10.1038/s41598-019-49743-4

**Published:** 2019-09-13

**Authors:** Anna-Sophia Pörings, Torsten Lowin, Bianca Dufner, Joachim Grifka, Rainer H. Straub

**Affiliations:** 1 Laboratory of Experimental Rheumatology and Neuroendocrine Immunology, Dept. of Internal Medicine, University Hospital Regensburg, Regensburg, Germany; 2 W. & B. Hiller Research Center of Rheumatology, Life Science Center, University Hospital Düsseldorf, Düsseldorf, Germany; 3 Department of Orthopedic Surgery, University Hospital Regensburg, Asklepios Clinic Bad Abbach, Bad Abbach, Germany

**Keywords:** Thyroid diseases, Rheumatoid arthritis

## Abstract

While patients with rheumatoid arthritis (RA) sometimes demonstrate thyroidal illness, the role of thyroid hormones in inflamed synovial tissue is unknown. This is relevant because thyroid hormones stimulate immunity, and local cells can regulate thyroid hormone levels by deiodinases (DIO). The study followed the hypothesis that elements of a thyroid hormone network exist in synovial tissue. In 12 patients with RA and 32 with osteoarthritis (OA), we used serum, synovial fluid, synovial tissue, and synovial fibroblasts (SF) in order to characterize the local thyroid hormone network using ELISAs, immunohistochemistry, imaging methods, tissue superfusion studies, cell-based ELISAs, flow cytometry, and whole genome expression profiling. Serum/synovial fluid thyroid hormone levels were similar in RA and OA (inclusion criteria: no thyroidal illness). The degradation product termed reverse triiodothyronine (reverse T3) was much lower in serum compared to synovial fluid indicating biodegradation of thyroid hormones in the synovial environment. Superfusion experiments with synovial tissue also demonstrated biodegradation, particularly in RA. Cellular membrane transporters of thyroid hormones, DIOs, and thyroid hormone receptors were present in tissue and SF. Density of cells positive for degrading DIOs were higher in RA than OA. TNF increased protein expression of degrading DIOs in RASF and OASF. Gene expression studies of RASF revealed insignificant gene regulation by bioactive T3. RA and OA synovial tissue/SF show a local thyroid hormone network. Thyroid hormones undergo strong biodegradation in synovium. While bioactive T3 does not influence SF gene expression, SF seem to have a relay function for thyroid hormones.

## Introduction

Autoimmunity in rheumatic diseases is often pervasive affecting different tissues. This is best exemplified in systemic lupus erythematosus^1^ or in AIRE deficiency of the autoimmune polyglandular syndrome type I (called APECED), diseases which affect many different tissues including the thyroid^2,3^. Similarly, patients with rheumatoid arthritis (RA) often demonstrate therapeutically relevant hypothyroidism already at disease onset, which can be a sign for autoimmune thyroiditis (Hashimoto or IgG4-related thyroid diseases Riedel)^4^. Furthermore, thyroxin substitution was associated with a two-fold increased risk to later develop RA with or without antibodies against cyclic citrullinated peptides (ACPA-positive or ACPA-negative RA)^5^. On the other hand, autoimmune thyroid disease is often accompanied by autoimmunity in other tissues^6,7^. In summary, there could be a link between rheumatic diseases and the thyroid gland.

Autoimmune involvement of the thyroid gland is highly probable when typical clinical symptoms, ultrasound signs, and autoantibodies are present in serum. Autoantibodies are directed either against thyroglobulin (in Hashimoto much more prevalent than in Graves’ disease), thyroid peroxidase (in Hashimoto much more prevalent than in Graves’ disease), or thyrotropin receptors (only in Graves’ disease)^8^. Laboratory signs are high serum thyrotropin (TSH) levels indicating hypothyroidism or suppressed levels as in Graves’ disease with hyperthyroidism^8^.

**Figure 1:**
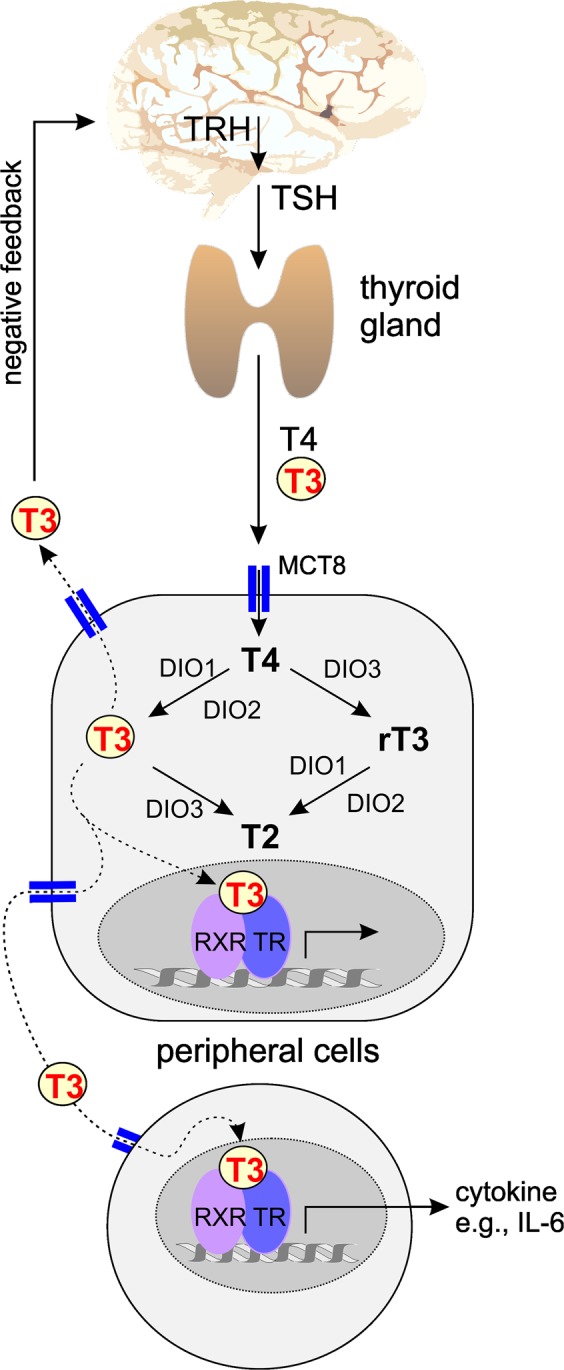
Schematic drawing of the thyroid hormone network. Thyrotropin (TSH) from the pituitary gland stimulates the thyroid to produce T3 and T4. In serum, these hormones are bound to transport proteins. Thyroid hormones enter cells via thyroid hormone transporters (MCT8, solute carrier family 16 member 2). Intracellularly, the biologically inactive T4 is converted to the biologically active T3 and to the degradation product reverse T3 (rT3). T3 and rT3 can be further converted to T2, which has no classical thyroid hormone activities. Conversion happens with deiodinases 1–3 (DIO). T3 exerts its action via the retinoic acid receptor (RXR) and the thyroid hormone receptor alpha and beta (TRα, TRβ). Abbreviations: T3, triiodothyronine; T4, thyroxine; TRH, thyrotropin releasing hormone.

While thyroid autoimmunity was often studied in different rheumatic diseases (e.g., refs.^4,5,9–20^), to the best of our knowledge, the synovial thyroid hormone network and a possible functional role of thyroid hormones has not been investigated in RA or osteoarthritis (OA) patients. Since rheumatologists usually do not know these pathways in detail, Fig. 1 describes the thyroid hormone network highlighting the biologically active triiodothyronine (T3).

Thyroid hormones have many typical proinflammatory functions. They can induce oxygen radical production in neutrophils^21^, advance IFN-γ-stimulated MHC class II expression^21,22^, stimulate IL-6, IL-8, and IL-12 secretion from different cell types^21,22^, support lymphocyte proliferation^21,22^, IFN-γ - stimulated natural killer cell activity^21,22^, and superoxide anion production^21–23^. Thyroid hormones are required for normal B cell development^24^.

Recent experimental studies have shown that down-regulation of the central part of the hypothalamic-pituitary-thyroid axis (HPT axis) observed during acute and chronic inflammation – called non-thyroidal illness syndrome with low free T3 - does not necessarily decrease thyroid hormone levels in key metabolic organs (summarized in ref.^25^). The differential regulation of local thyroid hormone availability mainly depends on expression of activating deiodinases (DIO1 or DIO2, see Fig. 1) and degrading deiodinases (DIO3, Fig. 1), which is tissue-specific^25,26^. Thus, one needs to look into cells typical for a target tissue, which has never been done in a rheumatic disease.

For example, during acute inflammation, in the muscle, the hormone-activating deiodinase DIO2 increases whereas the hormone-inactivating DIO3 decreases, which would lead to higher muscular T3 levels^25^. This is different in non-rheumatic chronic inflammation where DIO2 and DIO3 are elevated and degradation of bioactive T3 can be a necessary consequence^25^. A similar concept exists in the liver but similar pathways have not been described in synovial cells in arthritis^25^. In addition, hormone availability depends on presence of thyroid hormone membrane transporters, e.g., the monocarboxylate transporter 8 (MCT8; SLC16A2 solute carrier family 16 member 2)^27^. In target cells, respective thyroid hormone receptors (TRα and TRβ, both activating) are needed for functional effects (Fig. 1).

We hypothesized that the thyroid hormone network with its different elements exists in synovial tissue and fibroblasts in RA and OA. We further hypothesize that differences exist between cells from RA versus OA patients.

## Materials and Methods

### Patients

**Figure 2:**
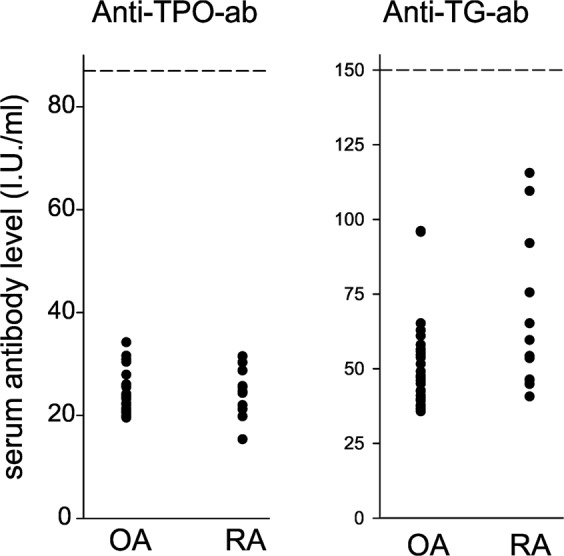
Serum levels of anti-thyroid peroxidase antibodies and anti-thyroglobulin antibodies in Patients with rheumatoid arthritis (RA) and osteoarthritis (OA) under study. Every point represents the mean value of two replicates of one patient. The dashed line represents the upper normal limit. The data show that patients did not demonstrate suspicious autoantibody levels indicative of autoimmune thyroid disease. The autoantibodies were measured by standard ELISAs (Cat. No. RE75511 and RE70951, IBL International, Hamburg). No statistical comparisons were carried out.

Material was obtained from RA and OA patients undergoing standard knee joint replacement surgery in the Department of Orthopedic Surgery. Patients with RA (n = 12) and OA (n = 32) were included, which did not demonstrate clinical or laboratory signs of autoimmune thyroid disease (for antibodies against thyroglobulin and thyroid peroxidase see Fig. 2). Table 1 shows the characteristics of these patients. During the process of surgery, we obtained peripheral blood, synovial fluid, and synovial tissue samples as described earlier^28^.

**Table 1:** Characteristics of patients under study. Data are given as means ± SEM, percentages in parentheses, and ranges in brackets.

	Osteoarthritis	Rheumatoid arthritis
Number	32	12
Age, yr	70.3 ± 1.3 [56–87]	66.7 ± 2.9 [51–82]^a^
Women/men, n (%)	17/15 (53/47)	8/4 (67/33)^b^
Disease duration, yr	n.d.	10.9 ± 1.1
C-reactive protein, mg/l	5.9 ± 1.7	28.7 ± 12.4*
Clinically Graves’ disease, n	none	none
Clinically IgG4-related thyroid disease Riedel, n	none	none
Clinically Hashimoto thyroiditis, n	none	none
**Comorbidities**		
Coronary heart disease	1 (3.1)	0 (0)
Depressive symptoms	2 (6.3)	0 (0)
Earlier embolic event	1 (3.1)	1 (8.3)
Glaucoma	2 (6.3)	0 (0)
Heart failure	13 (40.6)	4 (33.3)
Hyperlipidemia	11 (34.4)	2 (16.7)
Hypertension	20 (62.5)	6 (50)
Hyperurikemia	5 (15.6)	0 (0)
Osteoporosis	2 (6.3)	6 (50.0)**
Parkinsons disease	0 (0)	1 (8.3)
Prostatic hypertrophy, benigne	2 (6.3)	2 (16.7)
Type 2 diabetes mellitus	8 (25.0)	2 (16.7)
Urinary incontinence	2 (6.3)	0 (0)
***Medication***		
daily prednisolone, mg	n.a.	5.1 ± 0.8
prednisolone, n (%)	n.a.	10 (83)
methotrexate, n (%)	n.a.	10 (83)
leflunomide, n (%)	n.a.	1 (8)
sulfasalazine, n (%)	n.a.	1 (8)
hydroxychloroquine, n (%)	n.a.	0 (0)
biologicals, n (%)	n.a.	2 (17)
NSAIDs, n (%)	21 (66)	10 (83)
opioid analgesics, n (%)	8 (25)	2 (17)
proton pump inhibitors, n (%)	21 (66)	11 (92)
antihypertensive medication, n (%)	18 (56)	6 (50)
diuretics, n (%)	7 (22)	4 (33)
thyroxine, n (%)	0 (0)	0 (0)

^a^p = 0.335, ^b^p = 0.434, *p = 0.011, **p < 0.01. C-reactive protein was determined by standard laboratory techniques. Abbreviation: n.a., not applicable; n.d., not defined (the time point of first diagnosis is difficult to define); NSAIDs, non-steroidal antiinflammatory drugs.

RA patients fulfilled the American College of Rheumatology (formerly, the American Rheumatism Association) criteria^29^. OA patients were included as a control group with very little systemic inflammation (Table 1).

The Ethics Committee of the University of Regensburg approved the study (approval number 15-1 01-021). Patients knew the purpose of the study and gave informed consent. The procedures were in accordance with the ethical standards of the responsible committee on human experimentation and with the Helsinki Declaration of 1975, as revised in 1983.

### Measurement of thyroid hormones and TSH

Using ELISA, we measured free thyroxin (fT4), free triiodothyronine (fT3), reverse triiodothyronine (rT3), and TSH described by the suppliers (fT3: Cat. No. RE55231, detection limit: 0.05 pg/ml, IBL International, Hamburg, Germany; fT4: Cat. No. RE55241, detection limit: 0.05 ng/dl, IBL International; rT3: Cat. No. CEC022Ge, detection limit: 27.6 pg/ml, Cloud Clone Corp., Houston, TX; high sensitivity TSH: Cat. No. MP55011, detection limit: 0.015 µIU/ml, IBL International). In order to determine these hormones, we used serum (fT3, fT4, rT3, TSH), synovial fluid (fT3, fT4, rT3, TSH), and superfusate (see below; fT3, fT4, rT3, TSH). Hormone ratios were calculated using the picomolar concentrations of fT3, fT4, and rT3, and these ratios had no unit (pmol/pmol).

### Immunohistochemistry and cell density of positive cells

Of synovial tissue samples, 8 pieces of roughly 0.8 cm^2^ were used for immunohistochemistry. Samples were immediately placed in freezing medium (Tissue Tek, Sakura Finetek, Zoeterwoude, The Netherlands) and then quick-frozen in liquid nitrogen. Some samples were fixed in formalin and embedded in paraffin according to standard procedures.

Then, 6–8 μm sections were stained with a panel of antibodies directed against DIO1 (NPB1-19706, Novus Biologicals, via R&D Systems), DIO2 (ab135711, abcam, Cambridge, UK), DIO3 (NBP 1-05767, Novus Biologicals, via R&D Systems), MCT8 (thyroid hormone transporter, ab104689, abcam), TRα (ab42565, abcam), TRβ (ab5622, abcam), and trace amine associated receptor 1 (TA1, ab65633, abcam). Primary staining was visualized using respective secondary antibody conjugated to horseradish peroxidase (Dako, Glostrup, Denmark) and making use of the DAB substrate (Vector Laboratories, Burlingame, USA). In immunohistochemistry experiments, we controlled staining by using unspecific serum or unspecific IgG antibodies instead of primary antibodies and this constantly yielded negative result (see Figures).

The density of positive cells for DIO1, DIO2, and DIO3 was averaged from 17 randomly selected high-power fields at the magnification of 400x and expressed as cell number per square millimeter.

### Synovial tissue superfusion

One part of fresh synovial tissue was used for superfusion experiments as described earlier^28^. Eight pieces of about 16 mm^2^ were loaded into 8 superfusion chambers (80 µl volume). Synovial tissue was superfused with serum-free culture medium (RPMI 1640, 25 mM HEPES, 1% penicillin/streptomycin, 30 µM mercaptoethanol, 0.57 mM ascorbic acid, 1.3 mM calcium, all additions from Sigma, Munich, Germany). Superfusion was performed for 2 hr at 37 °C using a flow rate of 66 µl/min, superfusate was collected at 3 hours and immediately frozen, and stored at −80 °C until further use.

### Synovial tissue and synovial fibroblast preparation

One part of fresh synovial tissue was minced and put in dispase I (Roche Diagnostics, Mannheim, Germany). Digestion was carried out for at least 1 h at 37 °C on a shaking platform. The resulting suspension was filtered (70 µm) and spun at 300 g for 10 min. The pellet was then treated with erythrocyte lysis buffer (20,7 g NH_4_Cl, 1,97 g NH_4_HCO_3_, 0,09 g EDTA ad 1 l H_2_O) for 5 min and re-centrifuged for 10 min at 300 g. The pellet was resuspended in RPMI-1640 (Sigma Aldrich, St. Louis, USA) with 10% FCS. The cell number was calculated using a Neubauer cell counting chamber. A total of 100.000 cells were transferred to 75 cm² culture flasks. After overnight incubation, cells were supplemented with fresh medium. Synovial fibroblasts were obtained through culture conditions at passage 3 to 5.

### Cell-based ELISA

To study the cellular expression of DIO2, DIO3, TRα, and TRβ under proinflammatory conditions, 10.000 synovial fibroblasts were seeded in a 96-well plate (Nunc) per well and incubated with/without human recombinant TNF for 12 hours (10 ng/ml final concentration, Promocell, Heidelberg, Germany). Then, cells were fixed with 3.7% formalin (Merck, Hohenbrunn, Germany) in PBS for 20 min, and stored at 4 °C until the assay was performed. After blocking and permeabilizing cells in PBS with 0.3% Triton X-100 and 1%BSA for one hour, primary antibodies were added (final concentration 0.66 µg/ml for DIO-2 and DIO-3, 1 µg/ml for TRα and TRβ) in blocking buffer for one hour at room temperature. After washing, secondary poly HRP conjugated antibody (Poly HRP Goat Anti-Rabbit, Thermo Fisher Scientific Inc., Rockford, IL) was added (final concentration 1.25 µg/ml) for one hour at room temperature. Specific staining was detected by addition of TMB solution (Thermo Fisher Scientific Inc., Rockford, IL, USA). After stopping the enzymatic reaction, optical density was determined using an ELISA reader (Biorad, München, Germany).

### Stimulation of RA and OA synovial fibroblasts with T3, and gene expression profiling

In order to study a functional readout of synovial fibroblasts that was dependent on T3, synovial fibroblasts were incubated in 10^−7^ M T3. One million cells per well were seeded in 6 well plates in 3 ml RPMI-1640 medium without phenol red supplemented with 1% heat-inactivated fetal bovine serum, 2% 200 mM L-glutamine solution, 100 U/ml penicillin, 0.1 mg/ml streptomycin, 1% 1 M HEPES solution (all from Sigma), and 0.008 mg/ml ciprofloxacin (Fresenius Kabi, Bad Homburg, Germany). After 4 days of culture, cells were treated with 10^−7^ M T3.

Gene expression profiling of synovial fibroblast was carried out in the following way. After 6 h of treatment, cells were lysed and whole RNA was isolated using spin columns (Cat. No. 74104, RNeasy Mini Kit, Qiagen GmbH, Düsseldorf, Germany). Quantity and quality of whole RNA was tested with a Nanodrop 2000 Spectrophotometer (Thermo Scientific, Wilmington, USA) and a 2100 series Bioanalyzer (Agilent Technologies Inc., Santa Clara, USA), respectively. Affymetrix Gene Chip® Human Gene 2.0 ST arrays were performed on the respective sets of whole RNA (Cat. No. 902112, Thermo Scientific, Wilmington, USA). An authorized Affymetrix service provider analyzed the array (Kompetenzzentrum für Fluoreszente Bioanalytik, Regensburg, Germany).

### Flow cytometry for the detection of toll-like receptor 4 cellular surface expression (TLR4)

RA synovial fibroblasts from 4 donors were stimulated for 24 h or 48 h with 10^−7^ mol/l of T3 in RPMI medium containing 1% FCS. Then, cells were detached by accutase treatment (Sigma), washed and incubated with an anti-TLR4 antibody (Miltenyi, CD284, #130-100-378, 1:11). RA synovial fibroblasts were incubated for 10 min at 4 °C and then analyzed by flow cytometry (MACS Quant, Miltenyi).

### Statistical analyses

All data are given as mean ± SEM. Box plots give the 90th, 75th, 50th (median), 25th, and 10th percentile. From one individual patient, we obtained serum, synovial fluid, and tissue superfusate (see above) so that paired comparisons of hormone concentrations were possible. Paired data were compared using Wilcoxon signed rank test (SPSS/PC, Advanced Statistics, V22.0, SPSS Inc., Chicago). Group medians were compared by the non-parametric Mann-Whitney test (SPSS). In comparisons with a control level of 100% in experiments of TNF-stimulated regulation of key thyroid hormone targets, a Wilcoxon one-sample test was used (SPSS). Spearman rank correlation analysis was used to study the interrelation between two parameters (SPSS). In all graphs, we give the numbers of patients by demonstrating the black symbol for one patient. This allows the reader to estimate missing data. The above mentioned statistical tests were fed with the numbers shown in the graphs. Log2 signals in the gene expression analysis were compared by using students’ T-test for non-paired data. p < 0.05 was the significance level.

## Results

### Thyroid hormones in serum and synovial fluid

Serum levels of the pituitary hormone TSH were not different between OA and RA patients and remained in the normal range (1.42 ± 0.18 *versus* 1.16 ± 0.13 mIU/l; normal range: 0.35–3.5 mIU/l). Likewise, serum levels of the bioactive fT3 was similar between groups (OA: 2.2 ± 0.2 *versus* RA: 1.8 ± 0.2 pg/ml; 1.4–4.2 pg/ml), neither differed serum levels of the biologically inactive precursor fT4 (OA: 0.82 ± 0.04 *versus* RA: 0.78 ± 0.03 ng/dl; 0.8–2.0 ng/dl) and the degradation product rT3 (OA: 123.1 ± 16.3 *versus* RA: 162.9 ± 44.2 pg/ml; 100–240 pg/ml). In addition, we observed a comparable hormone ratio of serum fT3/serum fT4 indicating bioactivity (OA: 0.35 ± 0.03 *versus* RA: 0.28 ± 0.03, no unit) and a similar hormone ratio serum rT3/serum fT4 indicating biodegradation (OA: 18.9 ± 3.5 *versus* RA: 27.0 ± 7.6, no unit). These data indicate that both groups of patients had normal systemic thyroid hormone conditions and a normally functioning thyroid gland.

In synovial fluid, similarly comparable results were obtained in OA and RA patients for TSH (0.26 ± 0.04 *versus* 0.26 ± 0.05 mIU/l), fT4 (0.86 ± 0.05 *versus* 1.05 ± 0.12 ng/dl), rT3 (408.6 ± 35.0 *versus* 306.0 ± 55.8 pg/ml), and the hormone ratio rT3/fT4 indicating biodegradation (61.7 ± 8.0 *versus* 50.4 ± 12.1, no unit). In synovial fluid, fT3 was only measurable in 3 OA and 4 RA patients, and the values were near the detection limit of 0.05 pg/ml.

**Figure 3:**
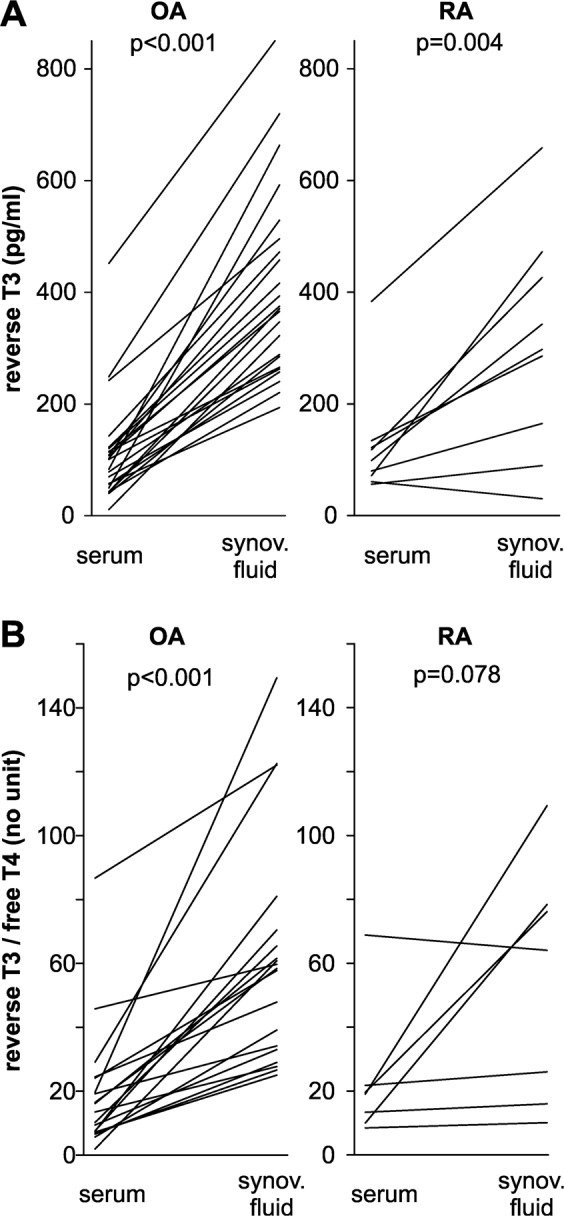
Levels of reverse T3 and ratio of reverse T3/free T4 in serum and synovial fluid. Every line represents the pair of values of one patient. The p-value for the comparison with Wilcoxon paired rank test is given. Abbreviations: OA, osteoarthritis; RA, rheumatoid arthritis; synov. fluid, synovial fluid; T3, triiodothyronine; T4, thyroxine.

**Figure 4:**
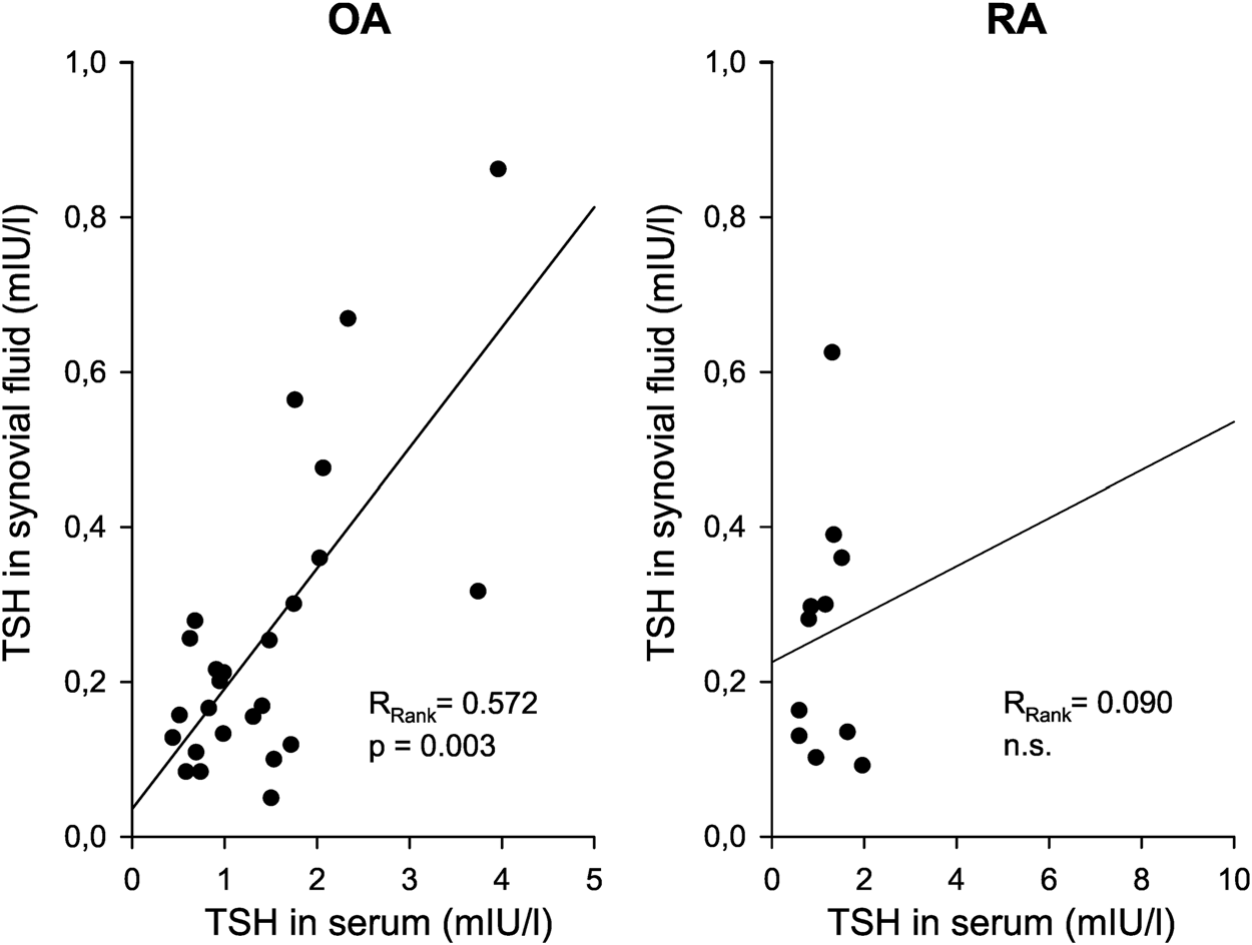
Interrelation between serum thyrotropin (TSH) and synovial fluid TSH. Every symbol in the graphs represent one individual patient with osteoarthritis (OA) and rheumatoid arthritis (RA). The linear regression line, the Spearman Rank correlation coefficient (R_Rank_), and the respective p-value are given.

In a direct pairwise comparison, separately in OA and in RA, rT3 was much lower in serum than in synovial fluid (Fig. 3). In OA only, the ratio of rT3/fT4 was lower in serum than in synovial fluid (Fig. 3). In RA, this also showed up in form of a trend (Fig. 3). This indicates a stronger biodegradation in the local synovial environment in both patient groups when compared to conditions in the blood. Interestingly, serum TSH was positively correlated with synovial TSH in OA patients (Fig. 4), which supports the concept that this hormone is part of the blood exudate with 10-fold lower synovial fluid levels. This did not reach the significance level in RA due to low numbers.

### Thyroid hormone converting enzymes in synovial tissue

**Figure 5:**
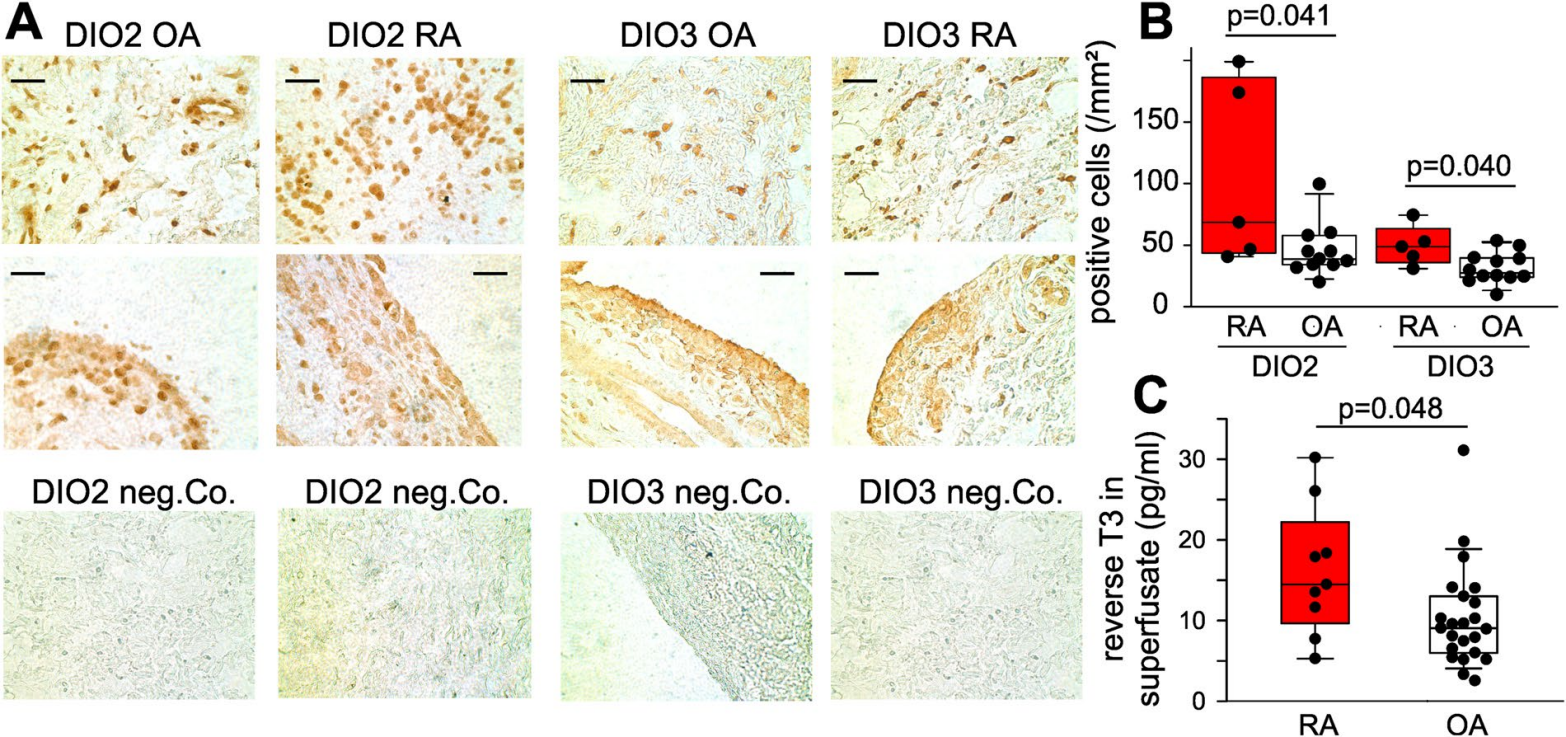
Synovial density of deiodinase (DIO)+ cells and levels of reverse T3 in synovial tissue superfusate of osteoarthritis and rheumatoid arthritis patients. (**A**) Immunohistochemistry of DIO2 and DIO3 including negative controls (neg. Co.). Scale bar = 60 µm. (**B**) Synovial density of DIO2+ and DIO3+ cells per mm². Every black symbol in B represents the mean value of 17 investigated synovial tissue high power fields of one patient. (**C**) Levels of reverse T3 in synovial tissue superfusate. Every black symbol in C represents the value of one patient. Box plots in (**B**,**C**) demonstrate the 10th (whisker), 25th, 50th (median), 75th, and 90th (whisker) percentile. For statistical comparisons in panel B and C, the Mann-Whitney-test was used. Abbreviations: OA, osteoarthritis; RA, rheumatoid arthritis; T3, triiodothyronine.

**Figure 6:**
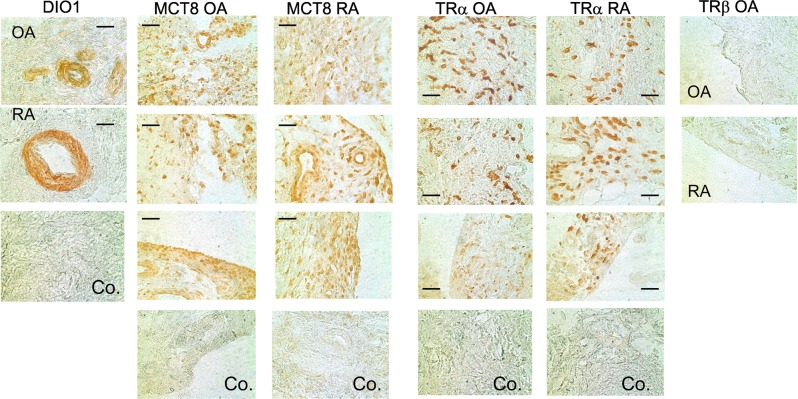
Synovial cells positive for deiodinase 1 (DIO1), the thyroid hormone transporter MCT8 (solute carrier family 16 member 2), thyroid hormone receptor alpha (TRα), and thyroid hormone receptor beta (TRβ). Scale bar = 60 µm. Abbreviations: Co., control staining; OA, osteoarthritis; RA, rheumatoid arthritis.

The biologically inactive thyroid hormones fT4 is converted to the biologically active fT3 (Fig. 1), which happens in target cells (called intracrinology). As shown in Fig. 1, DIO1 and DIO2 activate T4 to T3, while DIO3 deactivates T4 to rT3 and T3 to T2 (T2 has no classical thyroid hormone function). In synovial tissue, DIO2 and DIO3 are present in many cells of the lining layer and sublining area (Fig. 5). DIO1 is mainly present in vessel walls but does not exist in the lining layer or in sublining areas (Fig. 6, left column).

Importantly, cell density of DIO2-positive cells and DIO3-positive cells was higher in RA compared to OA synovial tissue (Fig. 5B). This indicates a higher turnover of T4 in RA synovial cells. In order to study this phenomenon in a functional way, we superfused freshly obtained synovial tissue and studied superfusate levels of rT3, the degradation product. In synovial superfusate, levels of rT3 were higher in RA compared to OA material (Fig. 5C), which supports the concept of stronger hormone degradation in RA compared to OA.

### Thyroid hormone transporter and receptors

Thyroid hormones enter cells through monocarboxylate transporters such as MCT8, encoded by the *slc16a2* gene. This transporter was present in synovial lining layers, sublining areas, and in synovial vessel walls of OA and RA patients (Fig. 6). This important prerequisite allows transport of T4 and T3 into local synovial cells.

In addition, function of the biologically active T3 depends on presence of either TRα or TRβ. In synovial tissue of patients with OA and RA, TRα is expressed (Fig. 6), but we failed to detect TRβ under these immunohistochemical conditions in complete synovial tissue (Fig. 6).

**Figure 7:**
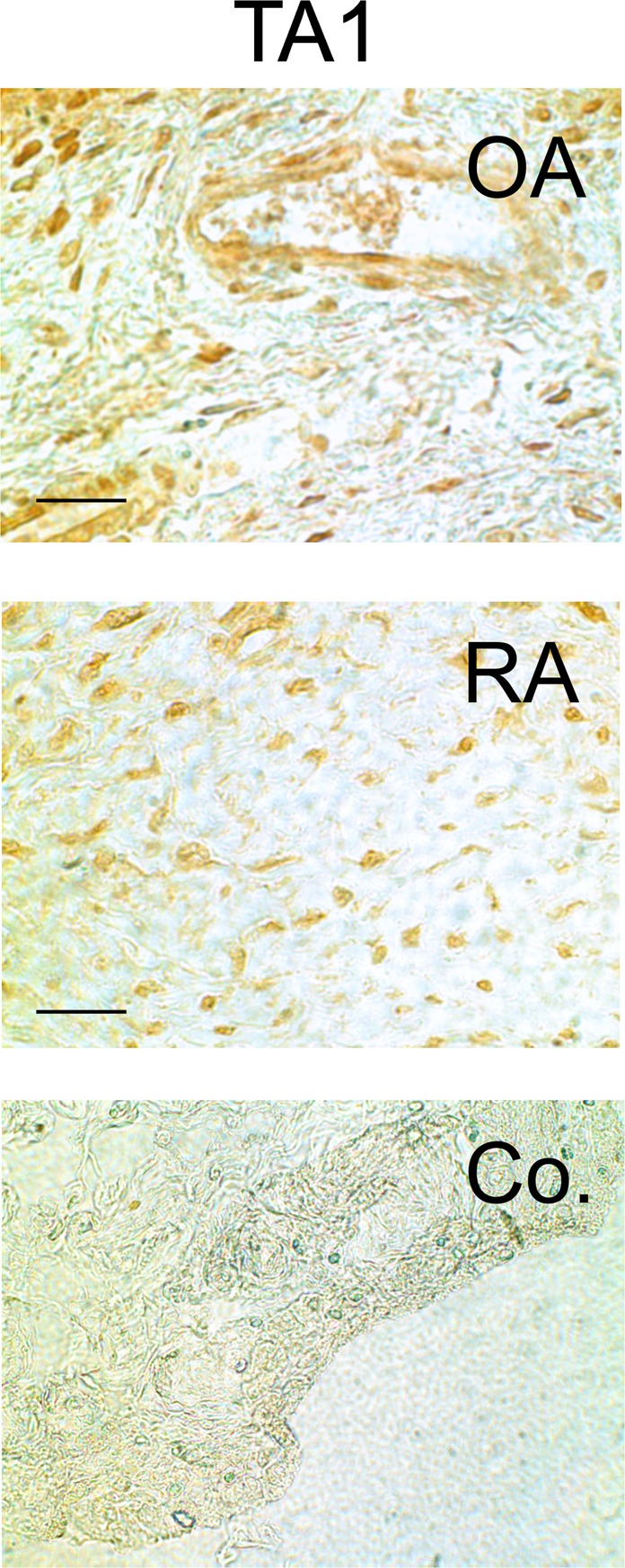
Immunohistochemistry of trace amine associated receptor 1 (TA1) in synovial tissue of a patient with osteoarthritis (OA) and rheumatoid arthritis (RA). The scale bar represents 60 µm. A control with unspecific immunoglobulin as the primary antibody is demonstrated.

For completeness, another candidate of downstream thyroid hormone signaling is the trace amine-associated receptor 1 encoded by the *TAAR1* gene. It is a G-protein coupled receptor that recognizes molecular derivatives of thyroid hormones such as thyronamines^30^. This receptor is ubiquitously present in synovial sublining tissue of RA and OA patients (Fig. 7).

### TNF-dependent stimulation of key thyroid hormone targets

**Figure 8:**
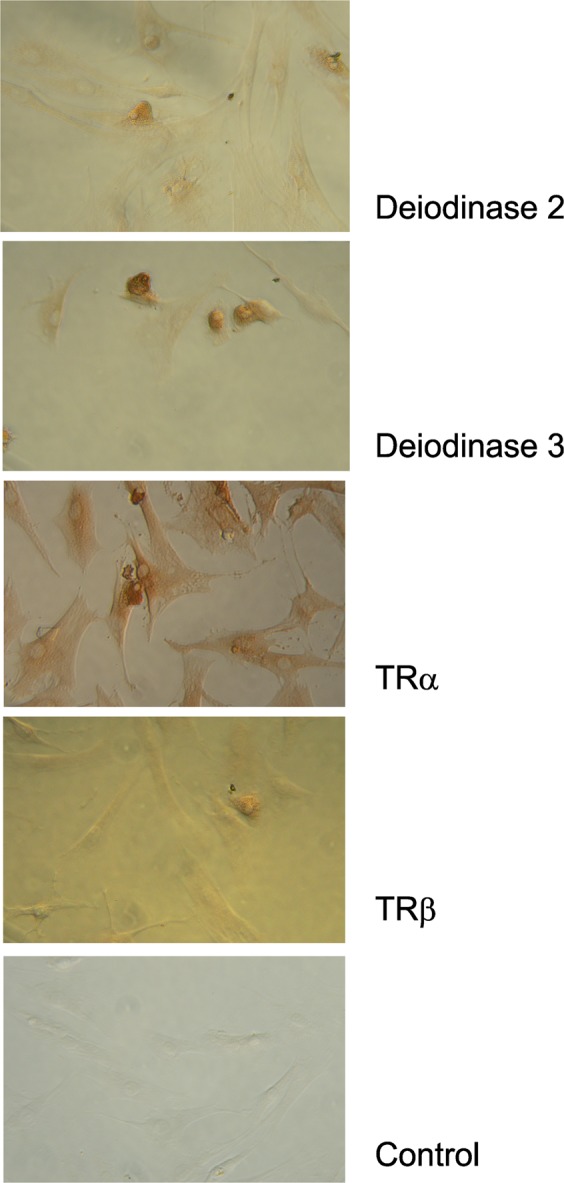
Immunocytochemistry of several markers of the thyroid hormone network in cultured synovial fibroblasts. The example of an RA patient is given. Abbreviations: TRα, thyroid hormone receptor type alpha; TRβ, thyroid hormone receptor type beta.

In order to study an influence of the proinflammatory cytokine TNF on several key targets of thyroid hormones, we first investigated presence of DIO2, DIO3, TRα, and TRβ in synovial fibroblasts after culture. All mentioned factors were present in synovial fibroblasts under culture conditions as demonstrated by immunocytochemistry including, this time also, TRβ (Fig. 8). However, TRβ staining was weak.

**Figure 9:**
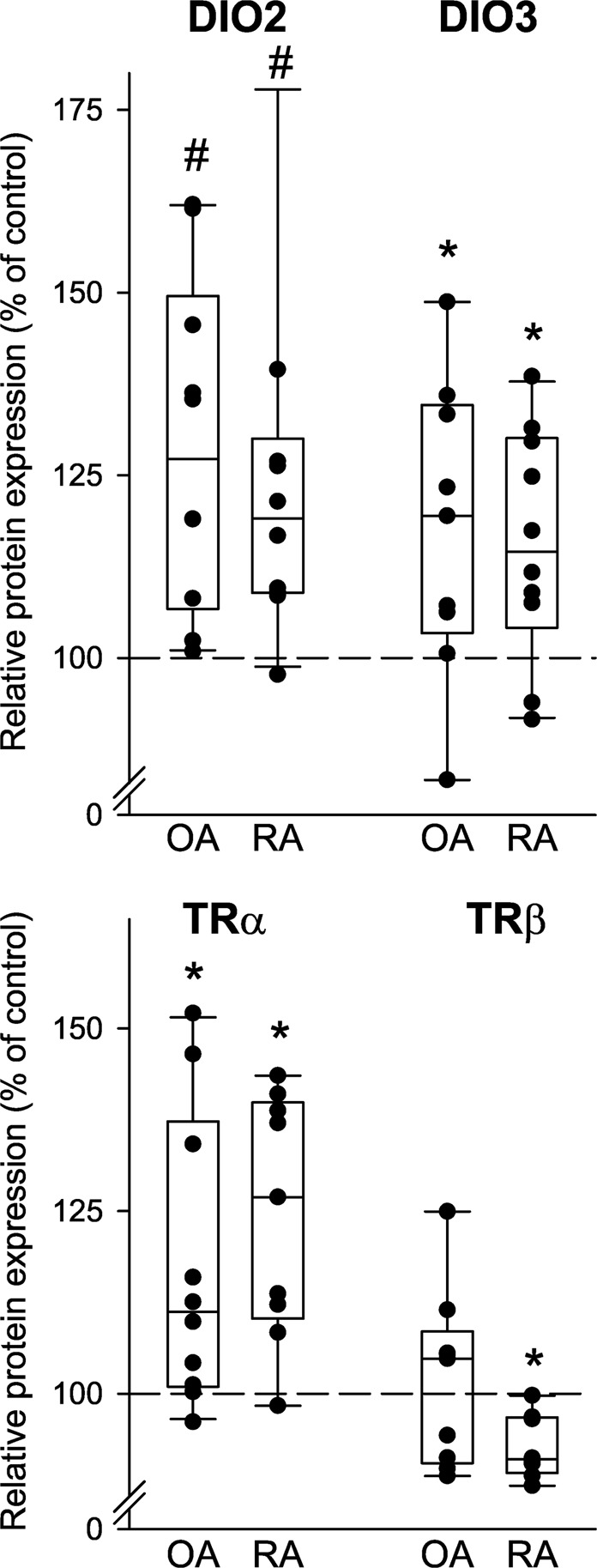
Modulation of protein expression of deiodinase 2 (DIO2), DIO3, thyroid hormone receptor alpha (TRα) and TRβ by TNF in synovial fibroblasts of patients with osteoarthritis (OA) and rheumatoid arthritis (RA). Box plots demonstrate the 10th (whisker), 25th, 50th (median), 75th, and 90th (whisker) percentile. Every black symbol represents the mean of duplicate values of one patient. The One-sample-Wilcoxon test was used for comparisons with the 100% control. *p < 0.05, #p < 0.008.

In cell cultures of synovial fibroblasts, TNF increased protein expression of DIO2, DIO3, and TRα in patients with OA and RA but decreased expression of TRβ in RA patients (Fig. 9). TNF had no influence on TRβ in OA patients (Fig. 9). This data indicate that inflammatory cytokines can influence local thyroid hormone handling and signaling.

### Influence of the active T3 on gene expression, TLR4 expression and IL-6 secretion of synovial fibroblasts

Finally, we wanted to study the direct influence of T3 on a readout parameter of synovial fibroblasts, which might be helpful for future studies. Using synovial fibroblasts of three RA patients, and using defined criteria (see bottom to Table 2), we detected three factors stimulated by T3 and two factors inhibited by T3 (Table 2). However, our criteria concerning the fold positive/negative change (±1,5 fold) and the p-value (<0.01) were weak, when one considers the huge amount of possibly regulated genes. Under more strict conditions, one would not have detected one single up- or do0wnregulated factor.

**Table 2:** Results from gene expression analysis of synovial fibroblasts stimulated with triiodothyronine (T3).

Symbol	Description	fold change	p-value	Accession number
SNORA22	Small nucleolar RNA SNORA22	2.57	0.008	ENST00000362701
MIR199B	microRNA 199b	2.38	0.007	NR_029619
TLR4	toll-like receptor 4	1.70	0.004	NM_003266
PWRN1	Prader-Willi region non-protein coding RNA 1	−1.53	0.009	NR_026646
SLC1A6	solute carrier family 1 (high affinity aspartate/glutamate transporter), member 6	−1.57	0.004	NM_001272087

A candidate gene is given in the table when the following criteria were fulfilled:

(1) Gene or nucleotide must exist in a database (accession number available).

(2) mRNA of pseudogenes, mRNA with undefined genome locations, and uncharacterized mRNA were excluded.

(3) The mean fold change of mRNA between control and T3 stimulation needed to be more than ±1.5-fold.

(4) The p-value for the comparison should be p < 0.01 (which is a weak p-value given the thousands of genes).

Positive (negative) values in mean fold change indicate higher (lower) mRNA expression of respective genes in cells stimulated (inhibited) with T3 compared to control. Whole RNA was isolated from synovial fibroblasts of 3 individual RA patients stimulated with T3 and synovial fibroblasts not stimulated (controls). Isolated whole RNA was analyzed with the Affymetrix GeneChip Human Gene 2.0 ST array.

Abbreviations: MIR, microRNA; NM, NCBI Reference Sequence for genes; NR, NCBI Reference Sequence for microRNA; RA, rheumatoid arthritis; TLR, toll-like receptor.

**Figure 10:**
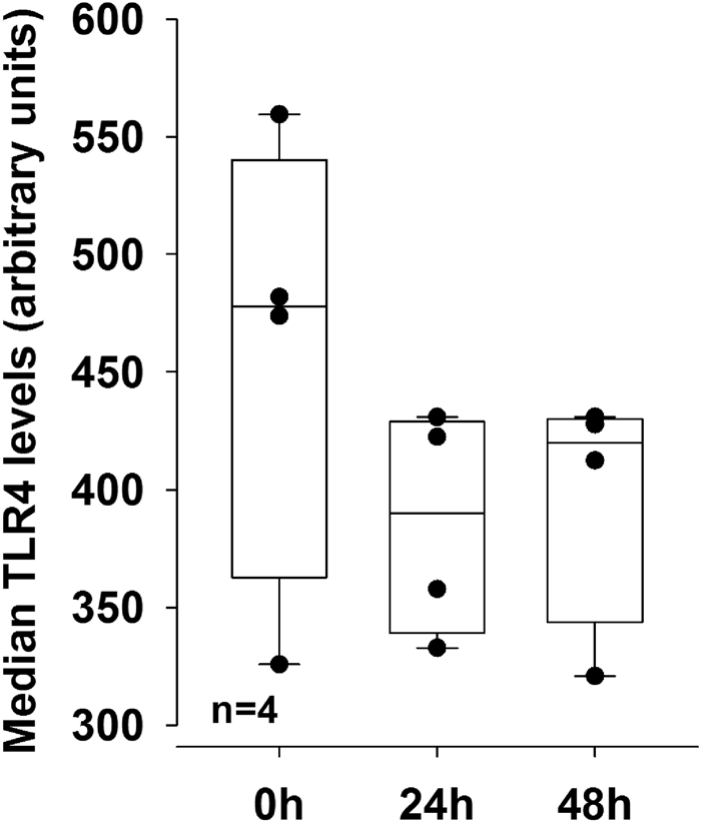
Median toll-like receptor 4 (TLR4) cellular surface expression on RA synovial fibroblasts. RA synovial fibroblasts were stimulated with 10^−7^ mol/l T3 over 24 and 48 hours. Surface expression of TLR4 was determined by flow cytometry analysis. Box plots demonstrate the 10th (whisker), 25th, 50th (median), 75th, and 90th (whisker) percentile. Every black symbol represents the mean of duplicate values of one patient (total: n = 4 patients). For comparisons of medians at 24 and 48 hours with the zero hour control (0 h), the Mann-Whitney-test was used.

Nevertheless, we studied one of these factors, which was toll-like receptor 4 cellular surface expression (TLR4). TLR4 is a marker with a direct immunological relevance due to normal appearance of endogenous TLR4 ligands that might trigger an inflammatory response in synovial fibroblasts of patients with RA or OA. As demonstrated in Fig. 10, stimulation of RA synovial fibroblasts with 10^−7^ mol/l T3 did not change the cellular surface expression after 24 and 48 hours of incubation. Other experiments with 10^−15^ to 10^−7^ M did not show any effects of T3 on other readout parameters like IL-6 (data not shown).

## Discussion

This study demonstrates a synovial thyroid hormone network in patients with RA and OA with systemically normal thyroid gland function. The synovial network includes the presence of thyroid hormones (T4, T3, rT3, TSH), thyroid hormone transporters (MCT8), thyroid hormone receptors (mainly TRα, but also TRβ in isolated synovial fibroblasts), thyroid hormone converting enzymes (the deiodinases 1 to 3), increased degradation of active hormone to rT3, and TNF-stimulated regulation of DIO2/3 and TRα, but surprisingly very few T3-regulated genes in synovial fibroblasts. These characteristics speak for a thyroid hormone network in the entire tissue. Synovial fibroblasts seem to be important in thyroid hormone activation and degradation but are inert towards T3. We have not studied T4 because T3 is the bioactive hormone.

The thyroid hormone system originates in the brain making use of thyrotropin-releasing hormone from the hypothalamus and TSH from the pituitary gland stimulating thyroid T4 and T3 release^8^. These two hormones feedback to the brain to inhibit central stimulation (Fig. 1). From a systemic perspective, in the circulation, T4 and T3 are acute stress hormones that influence metabolic and energy pathways^31–33^. Thyroid hormones accelerate metabolism, increase lipolysis, stimulate hepatic gluconeogenesis, induce thermogenesis, increase the Cori cycle (the cycle of glucose – lactate – glucose between liver and glucose-demanding tissue), decrease glycogen stores, accelerate insulin degradation, and increase GLUT4 glucose transporters in the skeletal muscle and monocytes^31–33^. Infusion of IL-6 into healthy volunteers increased fT4 for four hours, which is a sign of an acute stress response^34^. However, these effects were not long-lasting^34^.

With respect to the data on systemic thyroid hormones investigated in this study, we cannot see any difference between OA and RA patients, and these patients had normal systemic thyroid hormone function which a prerequisite of our study. Other studies also showed a normal systemic thyroid function in patients with RA and OA^35,36^. This might be clearly different in cases with parallel thyroid autoimmunity^4,5,9–20^, but these patients were excluded in our study. In addition, patients of our study showed a mild inflammatory situation as estimated by C-reactive protein.

While T4 is the inactive precursor of the biologically active T3, hormone availability depends on the local activation of T4 to obtain T3 in different tissues^37^. This is studied in metabolic organs like liver or skeletal muscles^25,37,38^. Conversion of thyroid hormones to other thyroid hormones depends on deiodinases expressed in target cells^39^. Such a system has many advantages because the active hormone is produced upon local demand. A similar phenomenon can be observed with glucocorticoids that can be activated or inactivated by 11β-hydroxysteroid dehydrogenases type 1/2 depending on local requirements^40^. It can also be observed with androgens and estrogens in synovial tissue, which depends on local TNF^41^. At present, it is unknown how thyroid hormones are processed in synovial tissue of healthy or arthritic patients.

In this study, we observed that deiodinases were present in synovial tissue, and RA patients showed more positive cells for DIO2 and DIO3 than OA patients, which is most probably responsible for a higher degradation of T4 and T3 to the degradation product rT3 (Fig. 2). This is more pronounced in RA compared to OA patients (Fig. 3C). This step plays an important role in the non-thyroidal illness syndrome with low fT3 levels, because peripheral degradation of T3 in inflamed tissue seems to play a decisive role^37,39^.

However, in RA and OA, we do not see the full picture of the non-thyroidal illness syndrome with low systemic levels of fT3 and fT4. Most probably, in our study, the local inflammatory situation is not strong enough to affect the rest of the body (e.g., the liver, the major converting organ). The question remains as to how low local levels of active T3 interfere with inflammatory signaling.

It became clear that the local thyroid hormone network can be regulated by TNF. In our study, TNF upregulates DIO2 and DIO3 which would support biodegradation of active T3. Others showed that IL-6 administration into healthy humans reduced T3 and increased rT3 levels 24 hours after infusion^34^. This indicates a rapid stimulated degradation of thyroid hormones by a proinflammatory cytokine^34^.

In activated neutrophils, DIO3 is also highly upregulated^25^. Here, activation of DIO3 does not only inactivate T4 and T3 but it also yields substantial amounts of elementary iodide within leukocytes^42^. Iodide can be a killing factor for bacteria, and DIO3 deficient mice have a much higher bacterial burden in lungs and spleen compared to wild type animals^25,43^. Thus, lack of DIO3 would impair bacterial clearance of the host. Since TNF is a major factor in bacterial clearance, TNF-upregulated DIO3 is a mechanism to fight bacterial infection. Now, it seems that this mechanism is used in synovial tissue of patients with RA and OA in the same way, but without the need to clear bacteria.

Our preliminary study of T3 effects in isolated synovial fibroblasts by gene expression profiling was surprising, because we expected many more T3-regulated genes. The observed effects are very weak given the enormous amount of investigated variables. Nevertheless, we studied cellular surface expression of toll-like receptor 4 (TLR4) on RA synovial fibroblasts. TLR4 is a good study candidate due to its role in inflam®mation and the presence of endogenous ligands to this receptor. However, TLR4 was not regulated by T3. It might well be that synovial fibroblasts are perfect in converting thyroid hormones to active an inactive forms, and that active thyroid hormones might play a role in neighboring cells such as macrophages. One might speculate that fibroblasts are only conversion machines while other cells in the neighborhood are affected.

The limitations of this study include the fact that we only investigated synovial fibroblasts. One might have added macrophages because they are the second most prevalent cell type in inflamed synovium. Similarly, T-and B cells and their subtypes might be a focus of extended studies. Co-culture experiments with two different cell types with or without physical cell separation can give a more complete picture of the thyroid hormone network. However, with every newly added cell type experiments become more complex and very time-consuming. In addition, other cell types might be more responsive towards bioactive T3 when compared to synovial fibroblasts. Due to space and time constraints, these experiments need to be carried out in future studies.

In conclusion, the synovial fibroblast can handle local thyroid hormones perhaps to serve a network. The local hormone situation is shifted towards inactive thyroid hormones like reverse T3, which is more marked in RA than OA patients. TNF is upregulating DIO3 that supports hormone degradation. Such a system is relevant in bacterial tissue infection, where it serves bacterial clearance, but it is similarly active in chronic inflammation like OA and more so in RA. This study gives a first idea of possible thyroid hormone control in an inflamed synovial environment in RA and OA patients.
